# Cerebrovascular disease associated with antiphospholipid antibodies: more questions than answers

**DOI:** 10.1186/1740-2557-3-3

**Published:** 2006-03-30

**Authors:** Carolyn Hawkins, Paul Gatenby, Roger Tuck, Gytis Danta, Colin Andrews

**Affiliations:** 1 Department of Clinical Immunology, The Canberra Hospital, Garran, Australia; 2 Department of Neurology, The Canberra Hospital, Garran, Australia; 3 Australian National University Medical School (ANUMS), Australian National University, Acton, Australia

## Abstract

Neurological syndromes occur in a significant number of patients with antiphospholipid antibodies. The optimal management for these patients however remains uncertain.

Our study is a descriptive analysis looking retrospectively at 45 patients who presented to the principal tertiary referral centre in the Australian Capital Territory, with either cerebral arterial or venous thrombosis for which there was no obvious cause for their presentation when initially reviewed. The diagnosis was based on the clinical findings made by one of three neurologists attached to our centre. Radiological findings and the presence of either IgM or IgG anticardiolipin antibodies, IgG anti-beta-2 glycoprotein 1 antibodies or a lupus anticoagulant were then documented.

In this group of patients three subgroups were identified:

1. Individuals that fulfilled the Sapporo Classification Criteria

2. Individuals with transiently positive antiphospholipid antibodies and

3. Individuals with persistently low positive antiphospholipid antibodies.

The most interesting of these three groups are those individuals with transiently positive antiphospholipid antibodies. A potential cause for presentation was identified in only one patient of this group with documented infective endocarditis and bacteraemia. Comparison with the other two groups suggested that there was little in terms of clinical presentation, radiological findings or intercurrent risk factors for thrombotic disease to distinguish between them. With disappearance of antiphospholipid antibodies, the individuals within this group have not had further thrombotic events.

Our observations emphasise the problems that continue to exist in relation to the occurrence of cerebrovascular disease in the context of antiphospholipid antibodies and the optimal management of these stratified groups. Our findings also raise an as yet unanswered question as to the signficance of these transiently positive antiphospholipid antibodies. In the absence of significant intercurrent risk factors our findings would suggest that in the group we describe that they are likely to be of clinical significance.

## Background

The antiphospholipid syndrome (APS) is an autoimmune disorder characterised by recurrent venous and arterial thromboses, recurrent foetal loss and spontaneous abortions, thrombocytopenia, and the presence of antiphospholipid antibodies [1,2]. While there is an association with other autoimmune diseases such as systemic lupus erythematosus, the condition may also arise independently of other autoimmune disorders [1,3,4]. The presence of antiphospholipid antibodies may also occur as associated epiphenomena related to intercurrent infections and in this setting are said to be less likely to be associated with the characteristic syndrome [5]. Furthermore, a number of individuals with high titre anticardiolipin antibodies do not develop APS and studies suggest that additional factors or a "second hit" are required to cause endothelial dysfunction and activation of a pro-coagulant phenotype [6,7].

Neurological syndromes occur in a significant proportion of individuals with antiphospholipid antibodies and include transient ischaemic attacks, stroke (either embolic or thrombotic), cerebral venous thrombosis, migraines, seizures, multi-infarct dementia and mononeuritis multiplex [2,8-10]. With strong evidence that appropriate treatment with anticoagulant therapy is effective in minimising both arterial and venous thrombotic disease in these individuals [11,12], it is important to be able to identify individuals with APS at the time of presentation. Classification, but not clinical diagnosis is assisted by the Sapporo criteria, a preliminary classification formulated in 1999. In these criteria, venous or arterial thrombotic vascular disease is essential. The criteria currently mandate repeating antibody tests after 6 weeks [13]. This presents a problem where an acute diagnosis has implications for initiating active therapy.

While a firm evidence base is accumulating for the management of venous thrombosis and recurrent pregnancy loss in APS, the same cannot be claimed for cerebrovascular disease, which remains a problematic area with a number of unanswered questions.

This study involved the descriptive analysis of all patients presenting to a single centre with either cerebral arterial or venous thrombosis where a cause could not clearly be identified at the time of presentation. The presence of IgM or IgG anticardiolipin (aCL) antibodies, IgG anti-beta-2-glycoprotein1 (anti-β_2_GP1) antibodies and/or a lupus anticoagulant and results of radiological imaging were documented.

This series of patients illustrates each of the above conundrums, and emphasises the difficulty in clear-cut decision-making.

## Methods

Patients were selected consecutively on the basis of admission to The Canberra Hospital, the principal tertiary referral hospital for the Australian Capital Territory and surrounding south east New South Wales, with a total population of approximately 500,000, between January 1, 1995 to May 31, 2003 inclusive, with clinical or radiological evidence of cerebral infarction and the presence of IgM or IgG aCL antibodies, IgG anti-β_2_GP1 antibodies (when this assay became routinely available at our centre in 2000) and/or lupus anticoagulant. Initial events were recorded back to 1975 and therefore in some individuals the diagnosis was made retrospectively, in that aCL were not routinely tested for until 1986. Currently, in this institution, patients are further investigated for the presence of prothrombotic factors including AT-III, protein C and protein S deficiencies in addition to prothrombin G20210A and Factor V Leiden mutations, where there are no identifiable causes for their presentation with either cerebral venous or arterial thrombosis. Over the study period January 1, 1995 to May 31, 2003, 1729 patients were admitted to The Canberra Hospital with cerebral infarction.

Following selection, clinical data were retrospectively and prospectively reviewed. For each patient the clinical findings were confirmed by examination by one of three neurologists. A number of other parameters were assessed including: intercurrent risk factors for vascular disease – hypertension, hyperlipidemia, tobacco use, obstructive sleep apnoea [14] – the presence of other procoagulant factors, the use of the combined oral contraceptive pill, a history of previous miscarriage, intrauterine growth retardation or pre-eclampsia and previous arterial or venous thrombotic events. Results of cerebral imaging, including computed tomography (CT) and magnetic resonance imaging (MRI), in addition to echocardiography and carotid duplex ultrasound, were collated. Follow-up antiphospholipid testing, ideally at least 6 weeks apart as defined in the Sapporo criteria [13] was collected where possible. Information regarding the therapy instituted at the time of diagnosis and long-term follow-up was obtained.

### Laboratory tests

IgM and IgG aCL antibodies were detected by a semi-quantitative enzyme-linked immunoabsorbent assay according to the manufacturer's instructions using a commercially available kit (Immuno Concepts RELISA, Sacramento, CA, USA). The test system is standardised using internationally recognised reference preparations obtained from the Antiphospholipid Standardisation Laboratory. Results were reported semi-quantitatively according to the manufacturer's instructions in standardised units as follows: negative (IgG, <8 G phospholipid units (GPL units), IgM, <8 M phospholipid units (MPL units); low positive (IgG, 8–20 GPL units; IgM 8–20 MPL units); medium positive (IgG, 20–80 GPL units; IgM 20–80 MPL units) and high positive (IgG, >80 GPL units; IgM, >80 MPL units). IgG anti-β_2_GP1 antibodies were also detected by a semi-quantative enzyme-linked assay according to the manufacturer's instructions using a commercially available kit (QUANTA Lite, San Diego, CA, USA). Results were reported semi-quantitatively in standard IgG anti-β_2_GP1 units (SGU) as follows: positive (IgG, > 8 SGU); The system was standardised using a set of reference calibrators available from the Rheumatology Laboratory, Seton Hall University, St Joseph's Hospital and Medical Centre (New Jersey, USA) and supplied with the commercial kit.

Lupus anticoagulants (LA) are a group of antibodies that interfere with phospholipid-dependent coagulation reactions. In the laboratory they are defined as prolonged clotting tests that are responsive to procoagulant phospholipids. By definition, the tests should not correct with the addition of normal plasma but are corrected by the addition of phospholipid. In our laboratory the diluted Russell's viper venom time (dRVVT) and kaolin clotting time (KCT) are used to detect LA [15]. The dRVVT is used as a screening test using a commercial kit (Gradipore, French's Forest, Australia). A mixing test is performed on all positive LA screen results, that is, the clotting time is 20% longer than the pooled platelet poor normal plasma (PNP). If the mixing test does not correct, a LA Confirm test is performed to confirm the presence of LA. The additional phospholipid in LA Confirm neutralises lupus anticoagulants to give a normal clotting time. The KCT is performed as per the method described in Gibson et al [16], where the difference in seconds between the neat pool (10:0) and the 8:2 mixture of PNP: patient is less than 20% different from the pooled normal plasma, the sample is considered negative. Where the difference is 20% or greater then the results are graphed on a six-point curve and the shape of the curve analysed. In the presence of LA the curve should be convex in the region near the Y-axis. A platelet neutralisation procedure is then performed to confirm the presence of a lupus anticoagulant. In this procedure a frozen-thawed platelet solution is added to the kaolin clotting time mixture to provide a source of platelet phospholipid able to overcome the effects of a lupus anticoagulant. With comparison to a normal pooled plasma clotting time, normalisation of the clotting time, confirms the presence of a lupus anticoagulant.

Antiphospholipid studies were repeated at least six weeks after the initial study. There have been no concerns regarding the performance of these assays in the routine quality assurance program for diagnostic laboratories in Australia run by the Royal College of Pathologists of Australasia (RCPA) and the National Association of Testing Authorities and conform with the consensus guidelines developed by the aCL Working Party comprising members of the Immunology Advisory, Quality Assurance and Scientific Education Committees of the RCPA [17].

## Results

Data were collected for 52 patients and 6 were immediately excluded because follow-up serology was not available. A further patient with infective endocarditis and therefore a potential cause for the presence of aCL was also excluded from further analysis. Of the remaining 45 the median age was 38.2 years with the age ranging from 6–63 years. There were 32 females and 13 males. Three groups of patients were identified on the basis of the results of antiphospholipid antibody testing.

In the first group of patients with primary antiphospholipid syndrome, 26 had completed strokes while seven of this group had had more than one stroke (two patients after anticoagulation with warfarin had been commenced). Multiple sites of the brain were involved and there was evidence for an embolic source in 8 patients. The clinical details for this group are summarised in Table 1. Twenty of the twenty-six patients were commenced on long-term high intensity warfarin therapy [11,12], either at the time of presentation or in the post-partum period. While some patients were originally diagnosed with obstetric antiphospholipid syndrome, their presentation was complicated by cerebral infarction or were noted on review during admission to have radiological evidence of past cerebral infarction. In those patients who had contraindications to the use of warfarin – three received low dose aspirin (100 mg daily), while the fourth individual was treated with clopidogrel at the standard dose (75 mg daily).

**Table 1:** Summary of clinical details for three groups of patients with aCL presenting with neurological events.

**Demographics**	**Patients fulfilling Sapporo classification criteria**	**Group with transiently positive aCL**	**Group with low positive aCL**
**Number of patients**	**N = 26**	**N = 7**	**N = 12**
**Median age (years)**	37.6 (6–63)	35.4 (16–51)	41.6 (19–59)
**Number of episodes/patient**			
1	19	4	6
2	7	3	6
**Clinical presentations**	**N = 33**	**N = 10**	**N = 18**
Superior sagittal sinus thrombosis	1	1	-
Higher centre function loss	3	1	1
Left hemiparesis/paraesthesia	10	5	6
Right hemiparesis/paraesthesia	6	-	2
Brainstem/cerebellar symptoms	5	-	5
Loss of consciousness/seizures	4	-	1
Fluent aphasia, right facial weakness	1	-	-
Bilateral cerebral infarction	-	1	-
Cranial nerve palsies	1	2	1
Right homonymous hemianopia	1	-	1
Transient ischaemic attacks	1	-	1
**Radiological findings**	**N = 26**	**N = 7**	**N = 12**
No abnormalities on CT or MRI scan	2	3	6
Left middle/posterior cerebral artery territory infarction	4	-	2
Right middle/posterior cerebral artery territory infarction	4	-	-
Low density area (left temporal lobe)	2	-	-
Increased signal cerebral deep white matter	3	1	2
Pontine/cerebellar infarction	2	1	1
Multiple cerebral infarcts	9	-	1
Superior sagittal sinus thrombosis	-	1	-
Bilateral narrowing of carotid syphons	-	1	-
**Antiphospholipid studies:**			
IgM aCL	6 moderate -high positive	-	3 low positive
IgG aCL	18 moderate -high positive	1 low positive, 1 moderate positive	6 low positive, 3 moderate positive
Lupus anticoagulant KCT	9	4	2
Lupus anticoagulant DRVVT	9	6	2
Low to moderate IgG anti-β_2_GP1antibodies	7/12 (data incomplete)	1 low positive	1 low positive
**Risk factors for vascular disease**			
Hypercholesterolaemia	3	-	4
Hypertension	4	1	3
Tobacco use	3	2	3
Oral contraceptive pill/hormone replacement therapy	6	-	1
Pregnancy/post-partum period	2	1	-
Heterozygosity for factor V Leiden mutation		1	-
**Long term warfarin**	19	2	-
**Warfarin (six months)**	-	1	1
**Low dose aspirin**	6	4	10
**Clopidogrel/ticlopidine**	1	-	1

Only four patients in this group have had further thrombotic episodes since warfarinisation. The four individuals were on long-term warfarin but in all four cases the INR had fallen to less than 3.0 in the period prior to their presentation with a thrombotic event. Three of these patients had additional arterial thrombotic events. One presented with both a stroke and pulmonary embolus, while two patients had further strokes and these have been included in the study. The last patient presented with a deep venous thrombosis involving the right lower limb. The duration of sub-therapeutic INR's prior to presentation in these individuals however could not be determined due to infrequent monitoring; however there have been no further recorded recurrences subsequent to these events.

The group with disappearing aCL comprised seven patients and details are summarised in Table 1. On repeat testing antiphospholipid antibodies could not be detected in any of these patients by all assays currently performed in our laboratory. Where possible these tests were performed at least six weeks after the initial tests, but in some cases were performed up to one year after initial testing. Repeated assays including LAC have remained negative and all of this group have been tested on four separate occasions albeit at differing time intervals. Only one in this group had a potential cause identified for the presentation with documented intercurrent infection. Streptococcus viridans was isolated from blood cultures and mitral valve vegetations demonstrated at echocardiography. This patient was therefore excluded from further analysis and is not included in the table. In this group, three patients received either low dose aspirin or clopidogrel where aspirin was contraindicated.

The third group comprised 12 patients with persistent low positive aCL antibodies on the basis of follow up testing at least six weeks after the initial testing and clinical data are summarised in Table 1. Within this particular group there were no clear distinguishing factors on the basis of the clinical presentation. Six of the twelve individuals experienced two distinct neurological events. Within the group of twelve, six had additional risk factors for vascular disease of which five had one or two associated risk factors for vascular disease, respectively. Four of the six individuals with additional risk factors had radiological evidence of completed strokes. The majority of individuals in this group have continued on low dose aspirin.

## Discussion

The three different groups of patients described emphasise a number of problems that exist in relation to the occurrence of cerebrovascular disease and antiphospholipid antibodies.

Current standard practice for patients with APS is to recommend indefinite anticoagulation with warfarin. This is derived from a limited experience with arterial thrombosis, a much larger and more definitive experience with venous thrombosis and the observations by many groups that these patients are prone to recurrent cerebrovascular events which, in principle, should be preventable [11,12]. In these studies venous and arterial disease is not clearly separated and comparison between different forms of recurrence prevention is limited [18].

The Antiphospholipid Antibody Stroke Study (APASS), a prospective cohort study within the Warfarin vs. Aspirin Recurrent Stroke Study (WARSS) does not provide answers for the management of the syndrome but challenges the current practice of long duration warfarin. However, the patients in this study were not representative of those described here, or in other series of cerebrovascular disease and APS. They were generally of advanced years, the aCL cut-off was much lower than the Sapporo criteria demand and the target INR of 2.0 lower than that recommended for APS. While the subgroup of patients with both LA and aCL had a greater risk for thrombo-occlusive events and appeared to derive benefit from warfarin, no other group did, nor did the patients overall [19]. Patients with embolic disease were excluded from WARSS and this study and most others provide little guidance on how to deal with the patients reported here, especially the problem of how to prevent recurrent ischaemic events. There is no guidance on how to approach a patient as a primary event and whether early diagnosis and management will make a difference to the ultimate outcome. Currently, the diagnosis is generally made after the critical cerebrovascular event is well under way, often recovering. To seriously consider how to manage these patients acutely the diagnosis would have to be appropriately acute as well.

The three groups here raise some very interesting questions. Twenty-six patients satisfy the Sapporo criteria and are quite similar to other such series of patients described previously. The range of antibody tests and the nature of the cerebrovascular lesions has the same broad spectrum as has been previously described [6,15]. Based on previous experience, steps need to be taken to reduce the recurrence rate and in this series warfarin was generally used unless contraindicated. This is in agreement with the practice of others [11,12,20] although there is no clear consensus as to the optimal treatment of these patients [21] with the findings of Crowther et al [22]challenging the need for high intensity anticoagulation, demonstrating that the absolute risk of recurrent thrombosis was low where the INR was maintained at 2.0–3.0. A more recent by Finazzi et al[23] reported similar results with high-intensity warfarin (INR 3.0 – 4.5) conferring no advantage over standard treatment (INR 2.0 – 3.0) in the prevention of recurrent thrombosis in individuals with antiphospholipid syndrome and was associated with an increased risk of minor haemorrhage. Both of these studies however, excluded patients with a history of recurrent thrombosis anticogulant therapy and therefore looked at a group likely to be a lower risk of recurrent events. In Crowther et al's study, individual's allocated to the high intensity group were below the target range 43% of the time. Three of the six individuals had an INR < 3.0 at the time of thrombosis while a fourth had ceased warfarin 137 days prior to the event [22] (reviewed in [24]). It is unclear from the second study as to how frequently those allocated to the high intensity group were sub-therapeutic but the mean INR of 3.2 would suggest that at least some individuals were sub-therapeutic throughout the time of the study. These studies therefore do not necessarily refute the findings of previous groups that high intensity warfarin may confer a benefit in those at high risk of recurrent thrombosis.

On the basis of the results of the Stroke Prevention in Reversible Ischemia Trial which found that high intensity anticoagulation (INR 3.0 – 4.5) was associated with an increased risk of major bleeding including cerebral haemorrhage [25,26], in addition to the small study by al-Sayegh [27] detailing haemorrhagic complications in a small group of patients with primary and secondary antiphospholipid syndrome, other groups have chosen to use low dose aspirin as prophylactic treatment in individuals with antiphospholipid syndrome who present with ischaemic stroke [28]. In our group however, 3 of the 8 patients developed further thrombotic disease while on low dose aspirin, two had recurrent strokes while the third had a spontaneous deep venous thrombosis. All three were subsequently treated with warfarin. However, it is difficult to draw conclusions based on on our study or those of al-Sayegh [27] and Derksen [28], due to their small size and lack of statistical power.

Patients in the second group at the time that the first laboratory tests became available could not be individually distinguished from those in the first group in contrast to observations in a group of patients with systemic lupus erythematosus where anti-β_2_GP1 antibodies were noted to drop at the time of thrombosis [29]. At the initial presentation, it would be reasonable to manage them in the same way, that is, anticoagulate, however with follow-up, this was probably unnecessary as the antibodies proved short-lived and the Sapporo classification criteria were not met. An alternative approach would be to wait in all patients until the Sapporo criteria are met – that is, defer a decision on definitive anticoagulation for six weeks. Given that the risk of recurrence is probably higher early on and community based studies suggest that the highest risk is in the first year with a mortality rate of 15–20% within the first 30 days following the initial stroke [30,31], this option is not seen as attractive. Either way, when faced with the patient, especially a young patient, with a cerebrovascular event and a criteria-positive antiphospholipid antibody there is clearly a dilemma. This subgroup which we believe has not been so clearly defined before would have to be considered in any future therapeutic study.

The third group also poses a problem and do not fulfill the classification criteria for antiphospholipid syndrome, as the antibody levels are insufficient to meet the Sapporo criteria. Those without radiological abnormalities might have had migraine equivalents, but the other patients, about half with definite radiological abnormalities and low level antibodies, had very few other risk factors. While two patients within this group were given six months of warfarin, the remainder were treated with low dose aspirin. None of these patients have had a recurrence to date, with a variable follow-up of 16 months -10 years. What indeed did they have and how should they be managed? Although in serological terms these patients resemble those of the APASS study [19], they are generally much younger. In the Stroke Prevention in Young Women Study [32], where the population studies included women aged between 15 and 44 years of age presenting with a first episode of ischaemic stroke, the presence of any anticardiolipin isotype or LA was associated with an increased risk of recurrent stroke with an overall relative odds ratio (OR) of 1.87 (95% CI, 1.24–2.83, P = 0.0027). However, antiphospholipid studies were only performed on one occasion and in some cases at a time distant to the stroke. The third group reported here is hard to directly compare to these patients, but could potentially overlap substantially with the patients described by Brey et al [32], where over half the patients had low positive antiphospholipid antibodies. Although excluded from the Sapporo criteria these patients would be important in any future study, warfarin was rarely used in patients in this group in our series.

This study has several limitations as it is essentially a descriptive study and is therefore open to bias given that the authors were not blinded when assessing the patients included in the study. The utility of the data is also hampered by a lack of standardisation in the approach to treatment. The assays used for assessing for the presence of aCL changed in 2000 and it is possible that some individuals may have become negative because a different assay was used. Additionally, in some individuals, the diagnosis was based on collection of retrospective data and there is no comparative group without antiphospholipid antibodies resulting in further sources of potential bias.

However, a strength of this study is that it represents what is actually done in a tertiary care centre in the face of conflicting evidence. We believe that such experience is worth reporting, reflecting as it does the realities of medical practice and serving as a useful adjunct to the gold standard randomised, double blinded trial.

We conclude that in our population, this combined retrospective and prospective analysis, confirms that recurrent events occur in young patients with cerebrovascular events and antiphospholipid antibodies. It does not provide information about the optimum method of preventing recurrence. Ultimately we believe there must be a much larger study of treatment and secondary prevention studies in stroke, with either emergency diagnosis of phospholipid antibody status, or a nested part of a much larger study with stratification of patients into at least the three categories described here.

## Competing interests

The author(s) declare that they have no competing interests.

## Authors' contributions

CH and PG were responsible for the initial concept, design and drafting of the manuscript. CH, PG, RT, GD and CA were involved in collation of patient data, the critical revision of the manuscript and intellectual input. All authors read and approved the final manuscript.
